# New Era of Studying RNA Secondary Structure and Its Influence on Gene Regulation in Plants

**DOI:** 10.3389/fpls.2018.00671

**Published:** 2018-05-22

**Authors:** Xiaofei Yang, Minglei Yang, Hongjing Deng, Yiliang Ding

**Affiliations:** ^1^Department of Cell and Developmental Biology, John Innes Centre, Norwich, United Kingdom; ^2^State Key Laboratory of Plant Genomics and National Center for Plant Gene Research, Institute of Genetics and Developmental Biology, Chinese Academy of Sciences, Beijing, China; ^3^College of Life Sciences, University of Chinese Academy of Sciences, Beijing, China

**Keywords:** RNA structurome, gene regulation, regulatory RNAs, RNA structure and function, plant RNA biology

## Abstract

The dynamic structure of RNA plays a central role in post-transcriptional regulation of gene expression such as RNA maturation, degradation, and translation. With the rise of next-generation sequencing, the study of RNA structure has been transformed from *in vitro* low-throughput RNA structure probing methods to *in vivo* high-throughput RNA structure profiling. The development of these methods enables incremental studies on the function of RNA structure to be performed, revealing new insights of novel regulatory mechanisms of RNA structure in plants. Genome-wide scale RNA structure profiling allows us to investigate general RNA structural features over 10s of 1000s of mRNAs and to compare RNA structuromes between plant species. Here, we provide a comprehensive and up-to-date overview of: (i) RNA structure probing methods; (ii) the biological functions of RNA structure; (iii) genome-wide RNA structural features corresponding to their regulatory mechanisms; and (iv) RNA structurome evolution in plants.

RNA secondary structure plays many essential roles in RNA synthesis, metabolism, and regulatory pathways (Bevilacqua et al., 2016; Vandivier et al., 2016). Previous efforts to determine RNA structure depended on classical and time-consuming techniques, such as nuclear magnetic resonance spectroscopy (NMR), X-ray crystallography, and cryo-electron microscopy (**Table 1**) (Lengyel et al., 2014). However, these methods yielded data limited to a few key RNAs with comparatively short length (less than 200 nt) and high abundance (∼1 μmol).

**Table 1:** RNA structure probing methods for both individual RNAs and at the genome-wide scale.

	Experiments	Capabilities	*In silico, in vitro*, or *in vivo*	Application	Reaction conditions	Reference
Targeted individual RNA structure probing	NMR; X-ray crystallography; Cryo-electron microscopy	Determine the three-dimensional structure at high resolution	*In vitro*	Ribosome, HIV	Specific buffer conditions	Lengyel et al., 2014
	Gel-based enzymatic and chemical probing	Determine the RNA secondary structure of high abundant RNAs up to 200 nt length	*In vitro* or *in vivo*	*E. coli*	Specific buffer conditions/cellular conditions	Noller and Chaires, 1972; Zaug and Cech, 1995
	SHAPE-CE	Determine the RNA secondary structure of high abundant RNAs up to 400 nt length	*In vitro*	*Arabidopsis*	Specific buffer conditions	Hawkes et al., 2016
	DMS/SHAPE-LMPCR	Determine the RNA secondary structure of low abundant RNAs	*In vivo*	*Arabidopsis*, human	Cellular conditions	Kwok et al., 2013
	SHAPE-Seq	Determine the RNA secondary structure of long RNAs	*In vitro*	RNase P pT181 sense RNA	Specific buffer conditions	Lucks et al., 2011
	SHAPE-MaP; DMS-MaP	Determine the RNA secondary structure of low abundant and long RNAs	*In vitro* or *in vivo*	TPP, HIV Yeast, human	Specific buffer conditions/cellular conditions	Siegfried et al., 2014; Smola et al., 2016; Zubradt et al., 2017
Genome-wide RNA structure profiling	FragSeq; PARS; PARTE; PIP-seq	Determine genome-wide *in vitro* RNA secondary structure with enzymatic probing	*In vitro*	Mouse Yeast, human *Arabidopsis*	Specific buffer conditions	Kertesz et al., 2010; Underwood et al., 2010; Li et al., 2012b; Wan et al., 2012, 2014; Gosai et al., 2015; Foley and Gregory, 2016
	CIRS-seq	Determine genome-wide *in vitro* RNA secondary structure with chemical probing	*In vitro*	Mouse	Specific buffer conditions	Incarnato et al., 2014
	DMS-seq; Structure-seq; Mod-seq	Determine genome-wide *in vivo* RNA secondary structure with chemical probing	*In vivo*	*Arabidopsis* Yeast *Oryza sativa*	Cellular conditions	Ding et al., 2014; Rouskin et al., 2014; Talkish et al., 2014; Deng et al., 2018
	icSHAPE	Determine genome-wide *in vivo* RNA secondary structure with chemical probing	*In vivo*	Mouse	Cellular conditions	Spitale et al., 2015

More recently, enzymatic and chemical structure probing methods have been developed to routinely and efficiently obtain structural information of individual RNAs. Ribonucleases (RNase) cleave either single-stranded (ss) RNA regions or double-stranded (ds) RNA regions to indicate RNA base-pairing status. The most commonly used enzymatic probing reagents include RNase V1 (for dsRNA), RNase S1 (for ssRNA), RNase A (for C/U in ssRNA), and RNase T1 (for G in ssRNA) (Knapp, 1989). The RNase-based RNA structure probing method has been used extensively in studying RNA structure with less toxicity, but with the limitation of cell permeability (Kwok, 2016). For chemical probing, two main types of chemical reagent can be used. One modifies the Watson-Crick base-pairing face on the nucleobase, as a direct measure of single-strandedness. Dimethyl sulfate (DMS) is one of the most commonly used nucleobase probing reagents as it easily penetrates the cell, a pre-requisite for *in vivo* chemical probing (Zaug and Cech, 1995). Another example is 1-cyclohexyl-(2-morpholinoethyl) carbodiimide metho-*p*-toluene sulfonate (CMCT), which targets the unpaired N3 position of uracil and the unpaired N1 position of guanine (Incarnato et al., 2014); while 3-ethoxy-1,1-dihydroxy-2-butanone (kethoxal) attacks the unpaired N1 and unpaired exocyclic amine positions of guanine (Noller and Chaires, 1972). Among these reagents, DMS is predominantly used to probe RNA structures in different organisms (Ding et al., 2014; Rouskin et al., 2014; Talkish et al., 2014; Deng et al., 2018). The other type of chemical reagent modifies the ribose, by selective 2′-hydroxyl acylation and which can be analyzed by primer extension (SHAPE) (Mortimer and Weeks, 2007; Spitale et al., 2013). A particular advantage of SHAPE is that it generates structural information for all four nucleotides at the same time.

Polyacrylamide gel electrophoresis (PAGE) assays were traditionally used to measure the modified pattern of both enzymatic and chemical reactions (Noller and Chaires, 1972; Knapp, 1989; Zaug and Cech, 1995). However, these gel-based assays were limited to highly abundant and short (less than 200 nt) RNAs. The application of capillary electrophoresis (CE) improved the detection limits of both the length (up to 400 nt) and the abundance of RNA (**Table 1**) (Watts et al., 2009). A recent application of CE on *Arabidopsis thaliana* long non-coding RNA (lncRNA), *COOLAIR*, revealed the remarkable complexity of RNA structure up to 750 nt (Hawkes et al., 2016). A further improved method on probing sensitivity, DMS/SHAPE-LMPCR, was developed in *Arabidopsis thaliana* (**Table 1**). This method achieved “attomole” sensitivity allowing RNA structure probing of low abundance RNAs in living cells (Kwok et al., 2013). By subsequently combining the action of DMS with next-generation sequencing high-depth RNA structural information of very long RNAs was achieved (Lucks et al., 2011; Smola et al., 2016). For instance, the structural information of over 18 kb lncRNA, *Xist*, was fulfilled in a single experiment (Smola et al., 2016). The development of these approaches has significantly improved the sensitivity and resolution for probing individual RNA structure both *in vitro* and *in vivo*. The capability for single nucleotide-resolution quantitative measurements on any RNA down to 1 attomole and up to 18 kb enables efficient functional investigation of RNA structure in biological processes.

Genome-wide RNA structure profiling was initially achieved by coupling enzymatic probing with next-generation sequencing, PARS (parallel analysis of RNA structure) (**Table 1**). It was developed in yeast by measuring the catalytic activity of two enzymes, RNase V1 (for dsRNA) and S1 (for ssRNA) (Kertesz et al., 2010). This method was extended in *Arabidopsis thaliana*, *Caenorhabditis elegans*, *Drosophila melanogaster*, and *Homo sapiens* (Li et al., 2012a,b; Wan et al., 2014). An enhanced method, PIP-seq (protein interaction profiling sequencing), complements RNA–protein interaction information with *in vitro* RNA structure profiling (**Table 1**) (Foley et al., 2015; Gosai et al., 2015; Foley and Gregory, 2016). A further improvement on genome-wide scale RNA structure profiling extended to living cells and addressed native RNA folding status. By harnessing the cell permeability of DMS, the first genome-wide *in vivo* RNA structure profiling method, Structure-seq, was developed in *Arabidopsis* (Ding et al., 2014, 2015) in parallel with DMS-seq and Mod-seq in yeast (Rouskin et al., 2014; Talkish et al., 2014) (**Table 1**). Both methods reveal *in vivo* RNA structures are more single-stranded than *in vitro* and *in silico* computational predicted RNA structures. Use of the Structure-seq method was recently extended to rice (Deng et al., 2018). A follow-up genome-wide *in vivo* RNA structure profiling method, icSHAPE (*in vivo* click SHAPE), was developed in mouse by using the SHAPE chemical reagent with the power of four-nucleotide probing (**Table 1**) (Spitale et al., 2015). In addition to measuring reverse transcription stopping, chemical modification can also be determined by mutational profiling (**Table 1**) (Siegfried et al., 2014; Smola et al., 2016; Zubradt et al., 2017).

These powerful genome-wide methods can provide an accurate and quantitative *in vivo* RNA structure map over tens of thousands of RNA with single nucleotide-resolution. These technological advances create an unprecedented scale for the in-depth study of the global impact of RNA structure in gene regulation. For example, regulatory RNAs are able to act as a master regulator in gene expression. In general, these regulatory RNAs directly turn on or off gene expression by altering RNA secondary structure. A recent study of RNA structure characterization on a range of regulatory RNAs in *Arabidopsis* is illustrated below (**Figure 1**).

**FIGURE 1:**
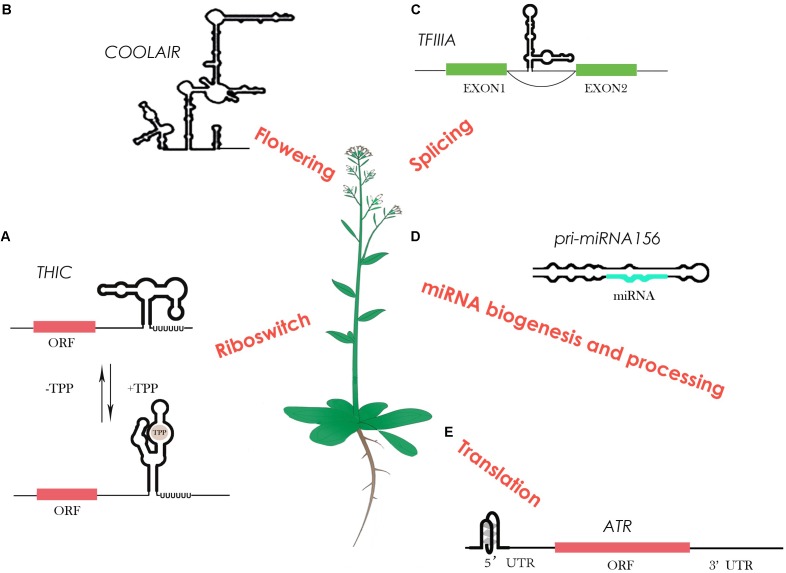
RNA structure characterization on regulatory RNAs in *Arabidopsis*. **(A)** The TPP (thiamin pyrophosphate) riboswitch in plants changes its own structure in response to different TPP concentrations, resulting in different 3′ end processing to control gene expression. **(B)** The highly-conserved plant lncRNA *COOLAIR* shows a highly complex structure that links to its biological function in flowering. **(C)** A 5S ribosomal RNA mimic regulates alternative splicing of transcription factor IIIA pre-mRNAs. **(D)** Several studies show that RNA structure determines miRNA biogenesis and processing. **(E)** An RNA G-quadruplex was reported to be able to regulate its own translation.

A riboswitch is a type of regulatory RNA that contains specific RNA structure segments, which can change conformation depending on specific ligand binding, e.g., metabolites. A well-studied example of a riboswitch is the vitamin B1 derivative thiamin pyrophosphate (TPP), which resides in the 3′ UTR region of the thiamin biosynthetic gene *THIC* (Wachter et al., 2007) (**Figure 1A**). With a low TPP concentration, the 3′ end processing of *THIC* mRNA results in a short 3′ UTR that permits high expression of the *THIC* gene. Conversely, with a high TPP concentration, TPP binds directly with the 3′ end of the RNA and induces a structural change that prevents splicing. This results in a long 3′ UTR inducing RNA degradation, subsequently reducing *THIC* gene expression (Wachter et al., 2007). Unlike riboswitches in bacteria that control translation through a structural change in the 5′ UTR, plants may have evolved a diverse and more complicated alternative 3′ end processing mechanism in order to cope with a large number of metabolites (Wachter et al., 2007).

Not only are some metabolites able to bind to specific RNA structures to regulate their synthesis pathways, but some are also able to regulate their own expression levels. A plant conserved pre-mRNA of *transcription factor IIIA* (*TFIIIA*) contains a 5S rRNA mimic structural element in one of its exons (Hammond et al., 2009) (**Figure 1C**). When ribosomal protein L5 binds to this 5S rRNA mimic, it triggers exon skipping in *TFIIIA* mRNA to control TFIIIA levels (Hammond et al., 2009). This ribosomal protein–mRNA interaction provides a new-found class of RNAs regulating alternative splicing to control the protein level.

Furthermore, specific RNA structural motifs such as G-quadruplexes (GQS) also play an important role in gene expression regulation. RNA GQSs are typically more stable in the presence of potassium or sodium. Tens of thousands of putative GQSs were identified in *Arabidopsis* and other plant species (Mullen et al., 2010). A recent study reported the first highly-conserved plant RNA GQS located in the 5′ UTR of *ATAXIA TELANGIECTASIA-MUTATED AND RAD3-RELATED* (*ATR*), inhibiting its translation when forming stable GQS structures (Kwok et al., 2015) (**Figure 1E**). Interestingly, potassium concentrations in plant cells can dramatically increase under drought stress (Mullen et al., 2010). Thus, GQS structural motifs in plants may specifically act as a regulator in response to abiotic stress, such as drought and salinity.

Long non-coding RNAs have also been shown as important regulatory RNAs involved in various biological processes. The study of lncRNA structures has been limited in the past due to their long length and low abundance. Advances in probing methods has enabled the highly-conserved plant lncRNA *COOLAIR* structure to be determined by chemical profiling with CE (Hawkes et al., 2016). *COOLAIR* is a key regulator of a major plant developmental gene *FLC* (*FLOWERING LOCUS C*), in response to vernalisation. The distal *COOLAIR* isoform in *Arabidopsis* (**Figure 1**) is highly-structured with numerous secondary structural motifs, an intricate multi-way junction, and two unusual asymmetric 5′ internal loops (Hawkes et al., 2016) (**Figure 1B**). Interestingly, a single nucleotide polymorphism (SNP) in the natural variation accession, Var2-6, is able to change the structure to affect the RNA stability, resulting in a late-flowering phenotype in Var2-6 (Hawkes et al., 2016). RNA secondary structure determination has progressed our understanding of the structure–function relationship of lncRNAs for the first time in plants.

The other well-known regulatory RNAs, miRNAs, also heavily rely on RNA structure for their regulatory functions (Herr and Baulcombe, 2004). The double-stranded region of miRNA precursors (pri-miRNAs) are recognized and processed by Dicer protein, an RNase III-like enzyme (Herr and Baulcombe, 2004). Previous studies in plants on both individual miRNA precursors and genome-wide assessment of pri-miRNA processing products confirmed that different structure determinants within pri-miRNAs compete for the processing machinery (Song et al., 2010; Bologna et al., 2013) (**Figure 1D**). A recent RNA structure characterization study by NMR shows the upper stem of a double-stranded region of pri-miR156 is important for Dicer processing at different temperatures, that substantiates the structure-determined Dicer processing feature (Kim et al., 2017). After Dicer processing, an Argonaute (AGO) protein will recognize the processed duplex miRNA to target mRNA containing complementary sequence for either RNA cleavage or translational inhibition (Valencia-Sanchez et al., 2006). Genome-wide *in vitro* RNA structure profiling in *Arabidopsis* revealed a less structured pattern in miRNA target sites that indicates the relationship between miRNA target efficiency and the single-stranded structural feature (Li et al., 2012b).

Apart from these studies on regulatory RNAs, recent genome-wide research also reveals the general role of mRNA structure in a variety of post-transcriptional regulations such as RNA maturation, RNA stability, RNA location and translation.

Alternative splicing is an important process in RNA maturation. More than 40% of *Arabidopsis* genes possess alternative spliced isoforms (Filichkin et al., 2010). The first *in vivo* RNA structure profiling in *Arabidopsis* revealed a significantly less structural pattern in the 40 nt region upstream of the 5′ splice site for unspliced events (including exon skipping and intron retention) (Ding et al., 2014). PIP-seq further revealed that this kind of structural pattern results in more RNA-protein interactions in *Arabidopsis* nuclei (Gosai et al., 2015; Foley and Gregory, 2016). Interestingly, PIP-seq also found the robust structure at the 3′ splice site is responsible for more protein interactions (Gosai et al., 2015; Foley and Gregory, 2016). Thus, these RNA structural features indicate an important role of RNA structure in regulating alternative splicing.

Another RNA maturation process is alternative polyadenylation (APA) that is found in over 60% of mRNAs in *Arabidopsis* (Loke et al., 2005). *In vivo* RNA structure profiling shows a strong structural pattern in the U- and A-rich upstream region of the cleavage site as well as a single-stranded region at the cleavage site (Ding et al., 2014). These patterns may correlate with the recognition of endonucleases for regulating APA. Further study using PIP-seq shows more protein bound up- and downstream of the APA cleavage site as compared to constitutive polyadenylation events (Gosai et al., 2015). However, APA sites do not exhibit altered *in vitro* RNA secondary structure compared to constitutive sites (Gosai et al., 2015). This suggests there may be different effects of RNA structure on both protein binding and cleavage activity, that warrant closer investigation.

In addition to RNA maturation, the relationship between RNA structure and RNA degradation has also been uncovered by *in vitro* RNA structurome analysis in *Arabidopsis*. Unlike yeast, highly-structured mRNAs are more likely to be degraded in *Arabidopsis*, probably via specific siRNA processing (Li et al., 2012b).

An interesting study on RNA mobility in plants shows that a stem-bulge-stem-loop tRNA-derived structural motif is sufficient to mediate mRNA transport. A large number of mRNAs containing this motif can be moved through graft junction (Zhang et al., 2016). Thus, RNA structure might also affect intercellular communication across plants.

Another major impact of RNA structure is its regulatory role in translation. Both *in vitro* and *in vivo* RNA structure profiling show a single-stranded region upstream of the start codon that might facilitate ribosome initiation (Li et al., 2012b; Ding et al., 2014). Moreover, a triplet periodic trend is observed in the CDS region but not in UTRs. These structure patterns are obvious in mRNAs with high translation efficiency and are absent in those with low translation efficiency (Ding et al., 2014). This implies that ribosomes may recognize RNA secondary structure as an additional layer of information alongside sequence content.

Additionally, RNA structure is also strongly associated with RNA methylation sites and RNA binding protein (RBP) sites. For example, N^6^-methylation of adenosine alters the stability of the A⋅U pair (Harcourt et al., 2017). Cellular RNAs show a decrease in base pairing around sites of m^6^A when they were methylated (Harcourt et al., 2017). Recent genome-wide studies indicate that the N^6^-methyladenosine (m^6^A) prefers single-stranded conformations rather than double-stranded structures (Zhao et al., 2017). A genome-wide study in *Arabidopsis* shows an enrichment of m^6^A around the start codon, stop codon and 3′ UTR region (Luo et al., 2014). Interestingly, this enrichment region of m^6^A is well-correlated with the single-stranded region identified in RNA structure profiling (Luo et al., 2014; Zhao et al., 2017). A study of the RNA structurome in rice also confirmed that higher m^6^A modification sites tend to have less RNA structure (Deng et al., 2018). This indicates that m^6^A association may alter RNA structure to more single-strandedness to facilitate gene regulation.

Another key player in post-transcriptional regulations is RBP. Unlike DNA binding protein, RBP associates not only with the primary sequence motifs, but also RNA structural patterns. A recent study combining genome-wide RBP profiling and RNA secondary structure profiling shows that RBP binding sites tend to be more single-stranded (Gosai et al., 2015). Interestingly, a nuclear PIP-seq study confirms that both RNA secondary structure and RBP binding sites show quite different patterns between hair and non-hair cells in plants (Foley et al., 2017). This suggests that cell-type-specific RNA structure and RBP binding may be a new regulatory mechanism during plant development.

From an evolutionary perspective, the conservation and diversity of RNA structurome between species remains poorly understood. A recent study compared, for the first time, the conservation and divergence of *in vivo* RNA structurome between plant species, to assess the evolutionary adaptation of RNA structure (Deng et al., 2018). This study found that *in vivo* RNA secondary structure conservation does not correlate with sequence conservation between rice and *Arabidopsis*. The conservation and divergence in both sequence and RNA secondary structure are highly relevant with specific biological processes (Deng et al., 2018). This indicates evolutionary selection not only modifies sequence, but also alters RNA structure to regulate gene expression. This in turn suggests that RNA secondary structure may serve a different layer of selection to sequence in plants.

Recent methodology advances have overcome previous limitations of both low-throughput and *in vitro* conditions for studying RNA secondary structure. These new methods, with single-nucleotide resolution, genome-wide scale and high sensitivity, significantly accelerate the study of *in vivo* RNA structure and associated biological functions. Plants are more sensitive than animals to varying environmental conditions, such as changes in temperature, salinity, acidity, and heavy metal concentrations (Schleuning et al., 2016). These factors are able to affect RNA folding (Tan and Chen, 2011; Wan et al., 2012; Sun et al., 2017). By applying these new RNA structure analysis methods under a range of environmental conditions, we will be able to determine how RNA structure alters in response to these changes. By integrating RNA structure profiling with mutagenesis assays and phenotypic analysis, the relationship between RNA structure and biological function can be investigated in greater details. Extending analyses to other plant species provides scope for exploring evolutionary selection at the RNA structure level. It is notable that this new era of studying RNA secondary structure provides unprecedented opportunity for discovering novel regulatory mechanisms of gene expression in plants.

## Author Contributions

XY, MY, HD, and YD wrote the manuscript. MY produced the figure. XY, HD, and YD made the corrections. All authors listed have made a substantial, direct and intellectual contribution to the work, and approved it for publication.

## Conflict of Interest Statement

The authors declare that the research was conducted in the absence of any commercial or financial relationships that could be construed as a potential conflict of interest.
